# Supplementary material for: The Rapid Evolution of Resistance to Vip3Aa Insecticidal Protein in Mythimna separata (Walker) Is Not Related to Altered Binding to Midgut Receptors
Source: Toxins (Basel). 2021 May 20;13(5):364. doi: 10.3390/toxins13050364 (PMC8190635; doi:10.3390/toxins13050364)
Supplement: Supplementary file 1 [file toxins-13-00364-s001.zip › toxins-1214319-supplementary.pdf]

Supplementary Materials: The Rapid Evolution of Resistance to Vip3Aa Insecticidal Protein in *Mythimna separata* (Walker) is not Related to Altered Binding to Midgut Receptors

Yudong Quan, Jing Yang, Yueqin Wang, Patricia Hernández-Martínez, Juan Ferré and Kanglai He

Table S1. The susceptibility of collected *Mythimna separata* against Vip3A and Cry1 toxins.

Toxins	Methods[1]	LC_{50} (95% FL)	LC_{95} (95% FL)	Slope ± SE	χ^2
Vip3Aa	μg/g	1.64 (0.55, 3.53)	35 (17, 148)	1.24 ± 0.13	10.0
	μg/cm^2	0.86 (0.29, 1.86)	185 (90, 779)	1.24 ± 0.15	11.1
Cry1Ab	μg/g	6.54 (2.44, 12.00)	289 (104, 3002)	0.96 ± 0.12	6.6
	μg/cm^2	0.16 (0.06, 0.29)	6.89 (2.49, 71.5)	0.60 ± 0.14	6.6
Cry1F	μg/g	26.1 (17.6, 37.5)	4700 (2043, 15,403)	0.20 ± 0.03	12.5
	μg/cm^2	1.71 (1.16, 2.46)	308 (134, 1010)	0.73 ± 0.07	10.0

[1] Methods: "μg/g" (toxin/diet) was performed by directly mixing the toxin and the diet as described previously [36]; "μg/cm^2" was obtained by the quantitative surface contamination assay [38].

Table S2. Response of *M. separata* to selection with Vip3Aa.

Generation	LC_{50} (95% FL) μg/cm^2	LC_{95} (95% FL) μg/cm^2	RR_{50} [a]	Slope ± SE	χ^2
Ms-S	0.86 (0.29, 1.86)	185 (90, 779)	/	1.24 ± 0.15	11.1
F1	26.8 (5.5, 84.7)	>4736	31	0.73 ± 0.16	4.4
F2	239 (106, 977)	>100,000	278	0.69 ± 0.11	17.9
Ms-S	0.81 (0.45, 1.42)	206 (76, 880)	/	0.68 ± 0.07	7.8
F5	224.9 (86.4, 816.8)	>500,000	277	0.48 ± 0.10	3.7
F6	>320	/	>400	/	/
F7	>1052	/	>1200	/	/
Ms-S	0.55 (0.26, 1.14)	213 (66, 850)	/	0.71 ± 0.07	7.8
F9	>1684	/	>3061	/	/

Table S3. Response of *M. separata* to selection with Cry1Ab.

Generation	LC_{50} (95% FL) μg/cm^2	LC_{95} (95% FL) μg/cm^2	RR_{50} [a]	Slope ± SE	χ^2
Ms-S	0.16 (0.06, 0.29)	6.89 (2.49, 71.5)	/	0.60 ± 0.14	6.6
F1	0.38 (0.17, 0.73)	91 (19, 2954)	2.5	0.69 ± 0.11	6.0
F2	0.19 (0.11, 0.30)	18.2 (7.9, 68.8)	1.2	0.83 ± 0.07	28.7
Ms-S	0.21 (0.15, 0.29)	3.25 (1.87, 7.78)	/	1.38 ± 0.60	4.8
F6	0.18 (0.12, 0.26)	4.69 (2.49, 12.04)	0.9	1.16 ± 0.13	0.6
Ms-S	0.11 (0.03, 0.25)	14.2 (4.04, 178)	/	0.78 ± 0.12	13.0
F7	0.28 (0.18, 0.42)	16.6 (7.43, 54.0)	2.5	0.93 ± 0.09	7.4
Ms-S	0.14 (0.08, 0.23)	6.65 (3.04, 20.8)	/	0.99 ± 0.11	5.9
F8	0.39 (0.20, 0.60)	49 (15, 139)	2.7	0.76 ± 0.08	6.9

Table S4. Response of *M. separata* to selection with Cry1F.

Generation	LC_{50} (95% FL) μg/cm^2	LC_{95} (95% FL) μg/cm^2	RR_{50} [a]	Slope ± SE	χ^2
Ms-S	1.71 (1.16, 2.46)	308 (134, 1010)	/	0.73 ± 0.07	10.0
F2	2.06 (0.66, 4.64)	236 (58, 6240)	1.2	0.80 ± 0.11	9.9
F4	5.63 (3.13, 12.8)	937 (174, 38,381)	3.3	0.74 ± 0.14	6.8
Ms-S	1.45 (0.32, 3.67)	62 (158, 9022)	/	0.63 ± 0.12	9.1
F7	10.6 (4.4, 18.3)	435 (160, 4375)	7.3	1.02 ± 0.22	0.6
F8	2.99 (1.82, 4.77)	134 (61, 440)	2.1	0.99 ± 0.10	10.6
Ms-S	1.73 (0.70, 4.25)	271 (55, 8540)	/	0.75 ± 0.10	13.9
F9	2.60 (1.77, 3.69)	60 (35, 125)	1.5	1.29 ± 0.12	5.1

[a] RR_{50} = Ms-R LC_{50}/Ms-S LC_{50}.

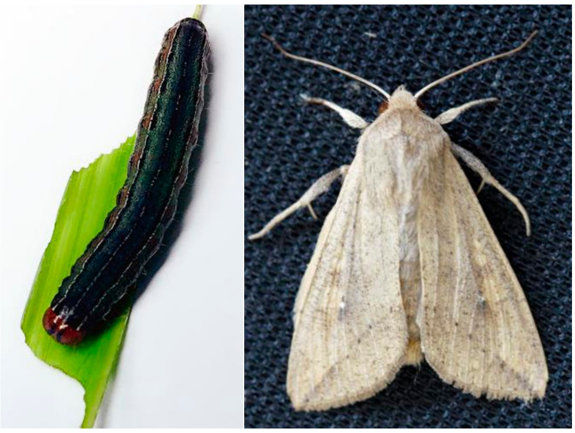

Figure S1. *Mythimna separata* larvae and adult.
